# Supplementary figures and images for: Glycine inhibits NINJ1 membrane clustering to suppress plasma membrane rupture in cell death
Source: eLife. 2022 Dec 5;11:e78609. doi: 10.7554/eLife.78609 (PMC9754625; doi:10.7554/eLife.78609)

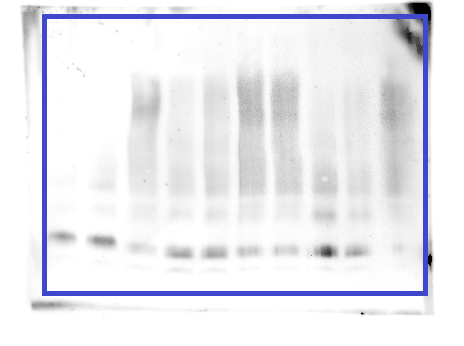

Supplement: Source data 1. [file elife-78609-data1.zip › WB TIFF images - source data - relevant bands/Figure 1 - figure supplement 1C - native NINJ1 - relevant bands.tif]

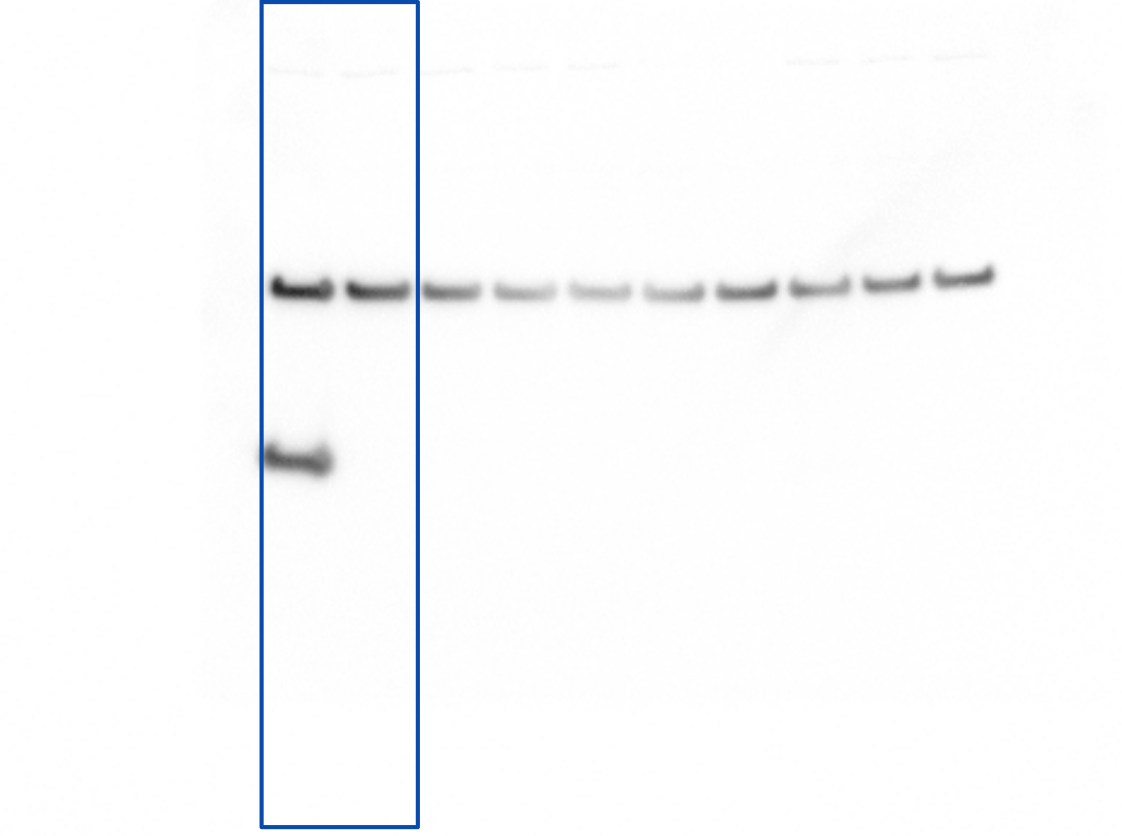

Supplement: Source data 1. [file elife-78609-data1.zip › WB TIFF images - source data - relevant bands/Figure 1 - figure supplement 2A - NINJ1 GAPDH - relevant bands.png]

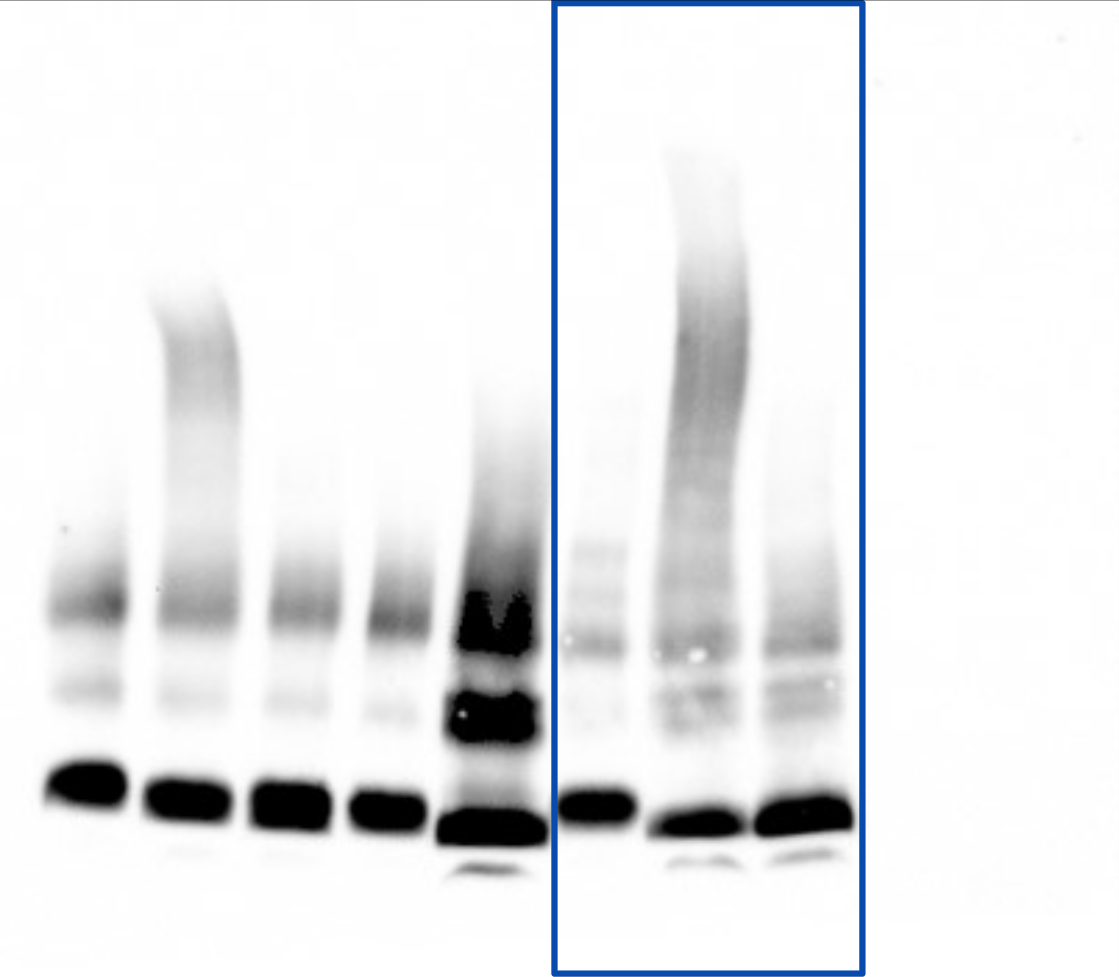

Supplement: Source data 1. [file elife-78609-data1.zip › WB TIFF images - source data - relevant bands/Figure 1 - figure supplement 2D - NINJ1 native - relevant bands.png]

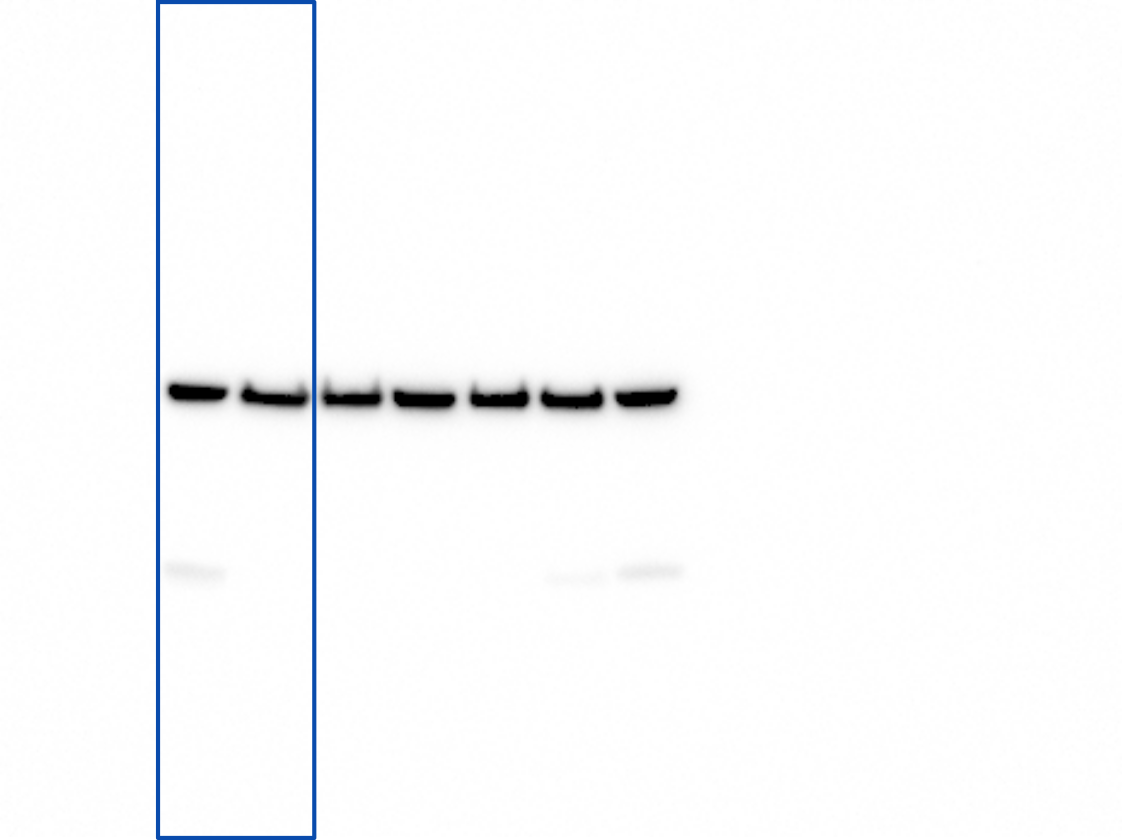

Supplement: Source data 1. [file elife-78609-data1.zip › WB TIFF images - source data - relevant bands/Figure 1A - iBMDM GAPDH - relevant bands.png]

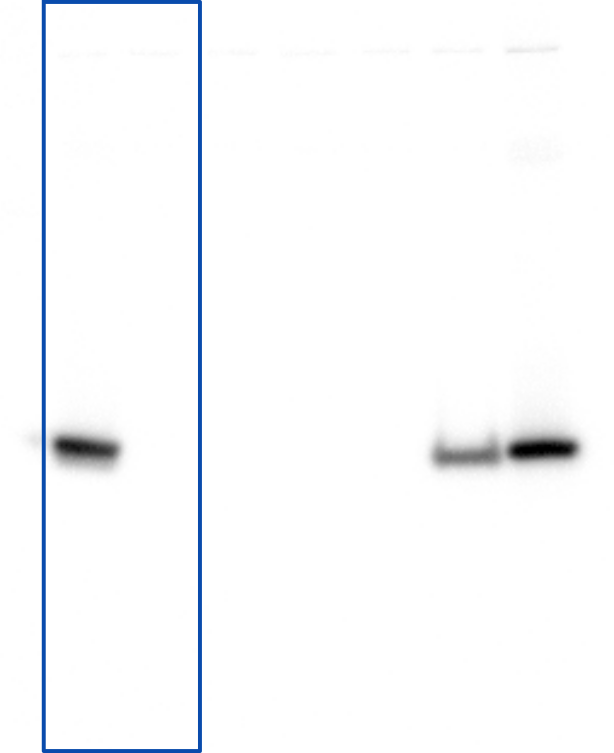

Supplement: Source data 1. [file elife-78609-data1.zip › WB TIFF images - source data - relevant bands/Figure 1A - iBMDM NINJ1 - relevant bands.png]

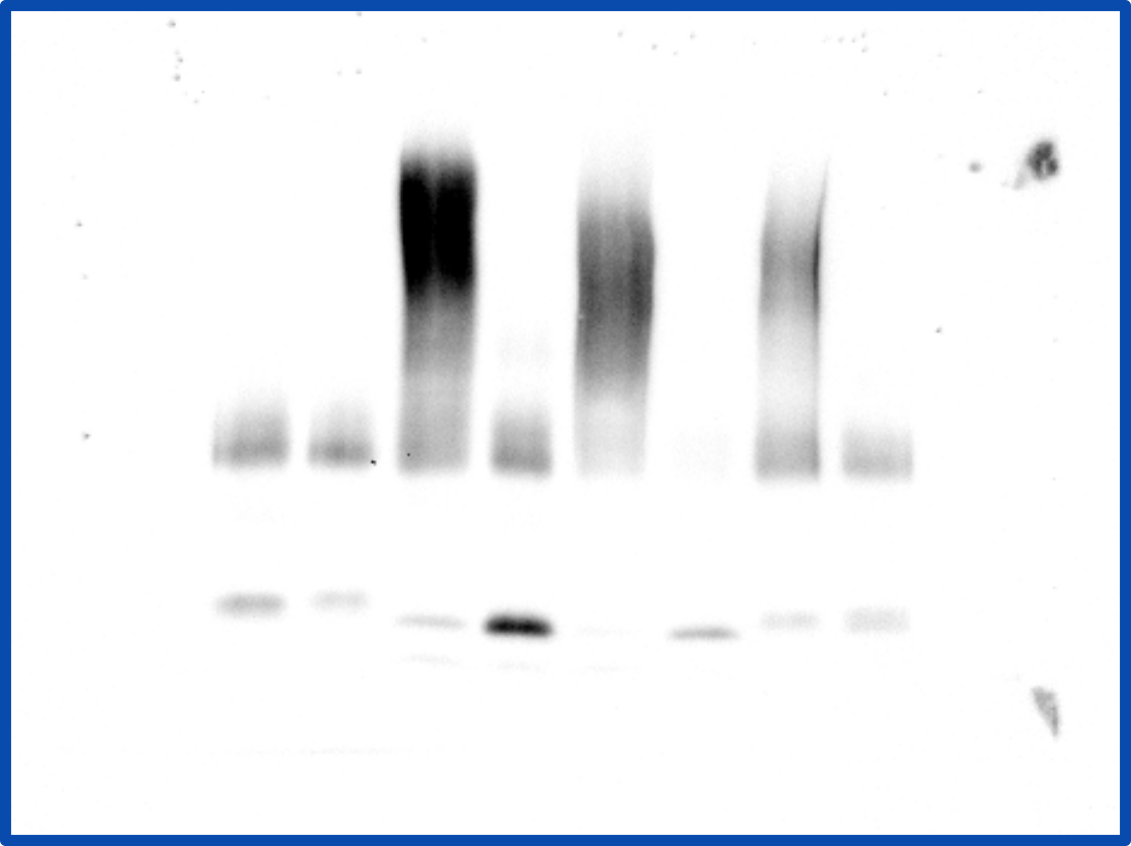

Supplement: Source data 1. [file elife-78609-data1.zip › WB TIFF images - source data - relevant bands/Figure 3A - native NINJ1 - relevant bands.png]

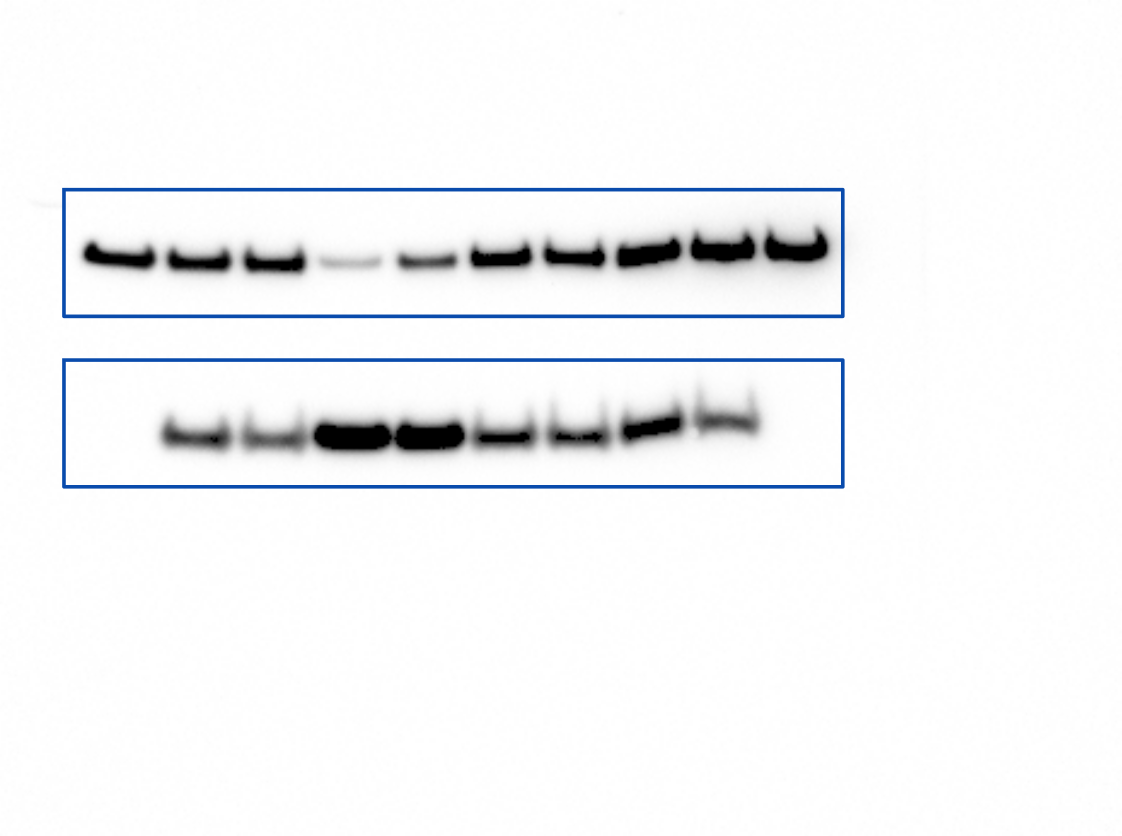

Supplement: Source data 1. [file elife-78609-data1.zip › WB TIFF images - source data - relevant bands/Figure 3A - SDS NINJ1 GAPDH - relevant bands.png]

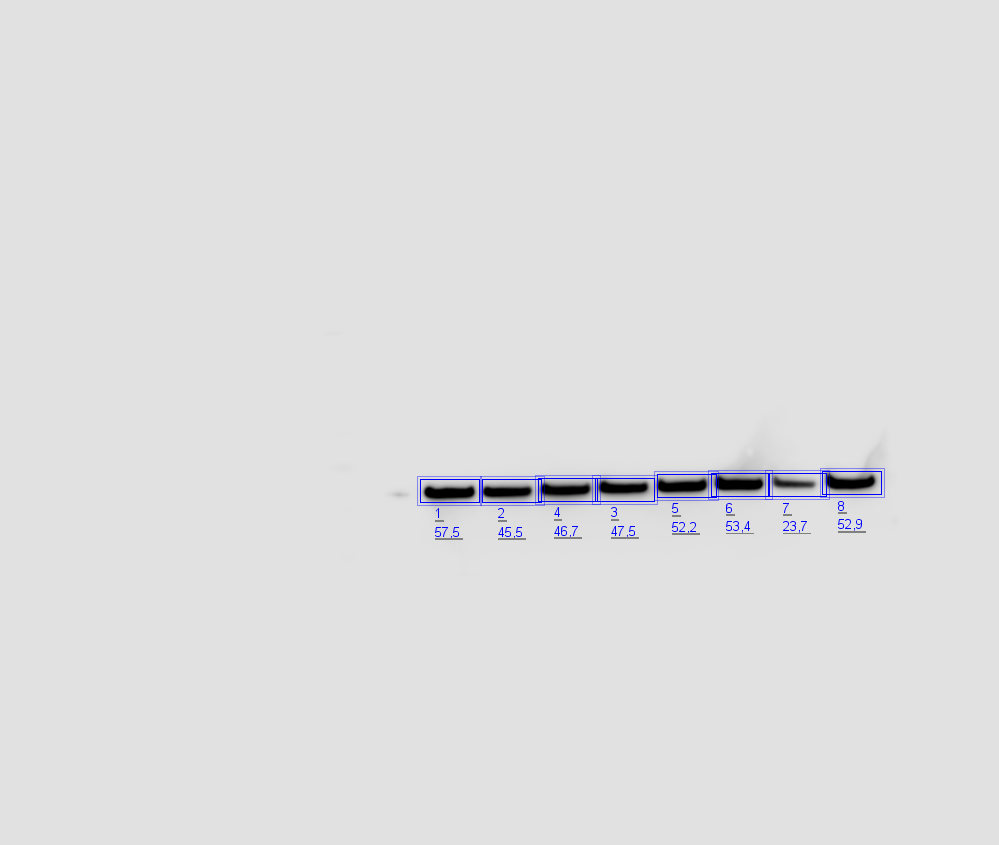

Supplement: Source data 1. [file elife-78609-data1.zip › WB TIFF images - source data - relevant bands/Figure 4 - figure supplement 2A - actin - relevant bands with quantification.tif]

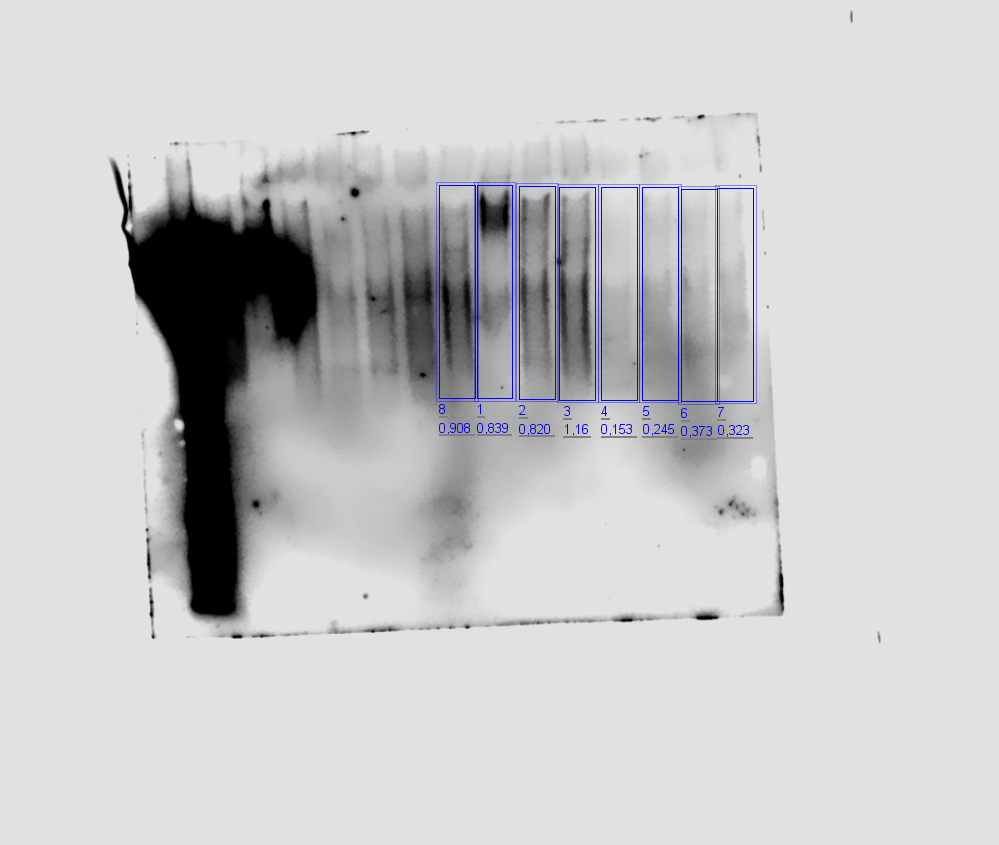

Supplement: Source data 1. [file elife-78609-data1.zip › WB TIFF images - source data - relevant bands/Figure 4 - figure supplement 2A - NINJ1 - relevant bands with quantification.tif]

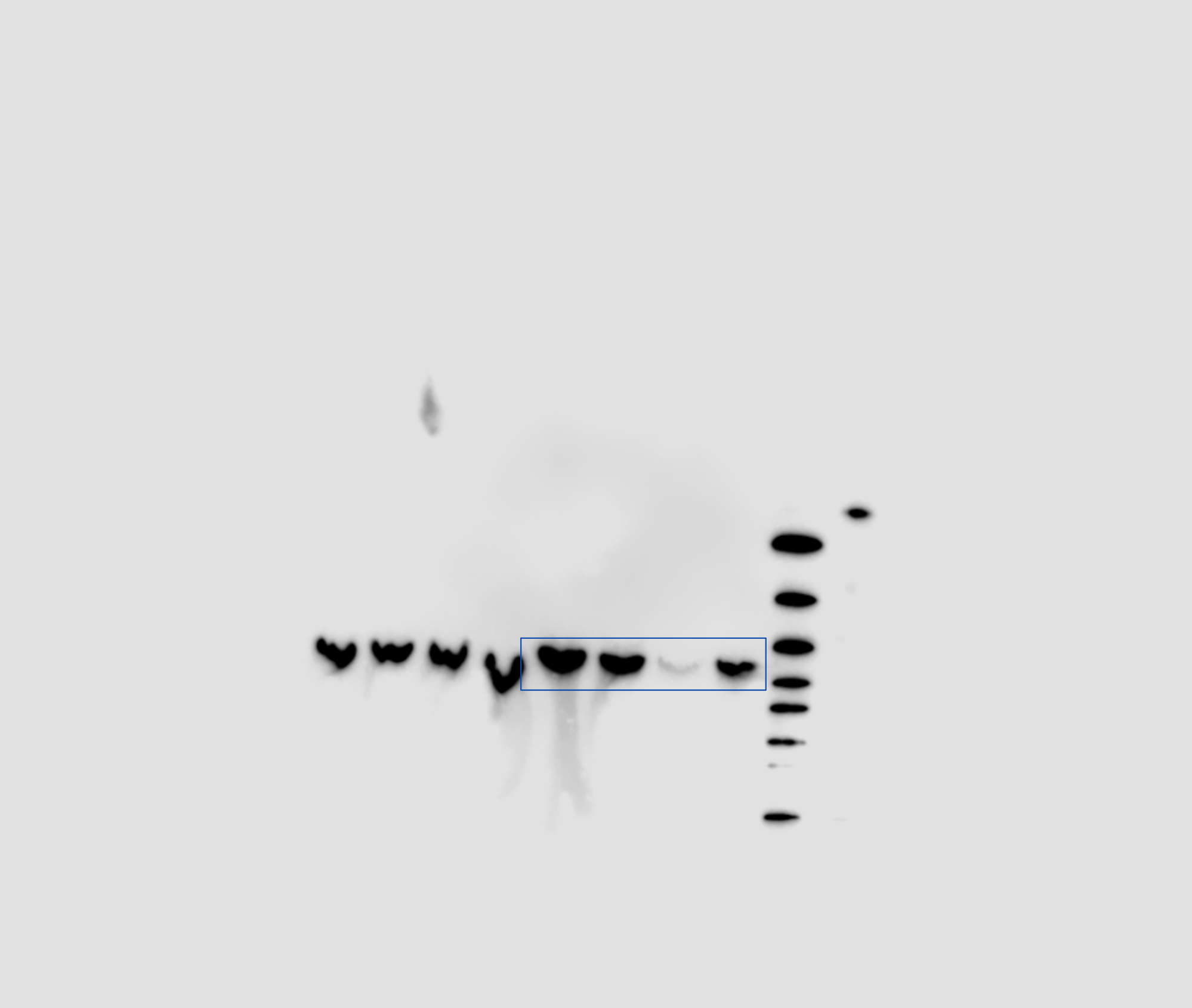

Supplement: Source data 1. [file elife-78609-data1.zip › WB TIFF images - source data - relevant bands/Figure 4 - figure supplement 2B - actin SDS - relevant bands.png]

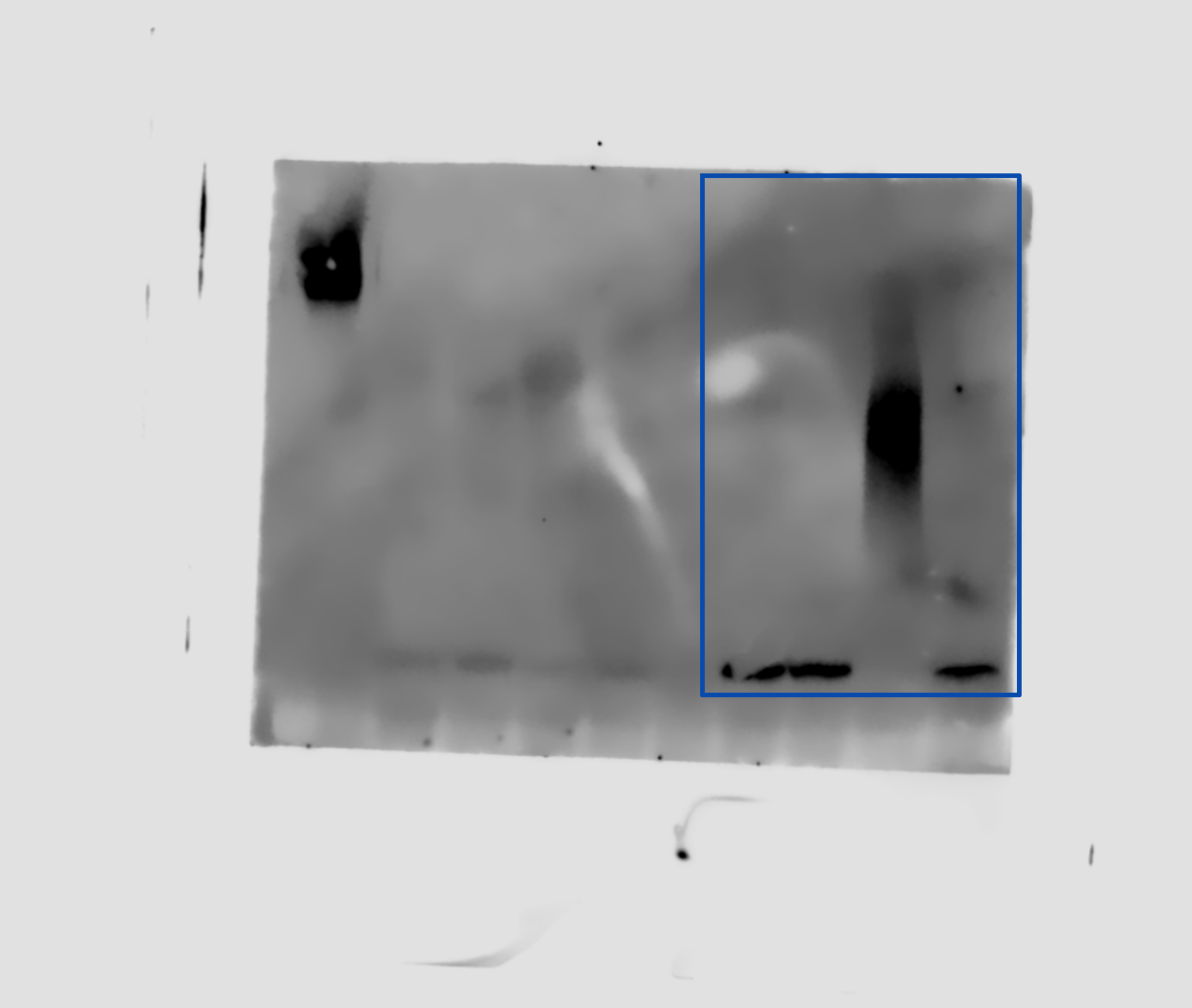

Supplement: Source data 1. [file elife-78609-data1.zip › WB TIFF images - source data - relevant bands/Figure 4 - figure supplement 2B - native NINJ1 - relevant bands.png]

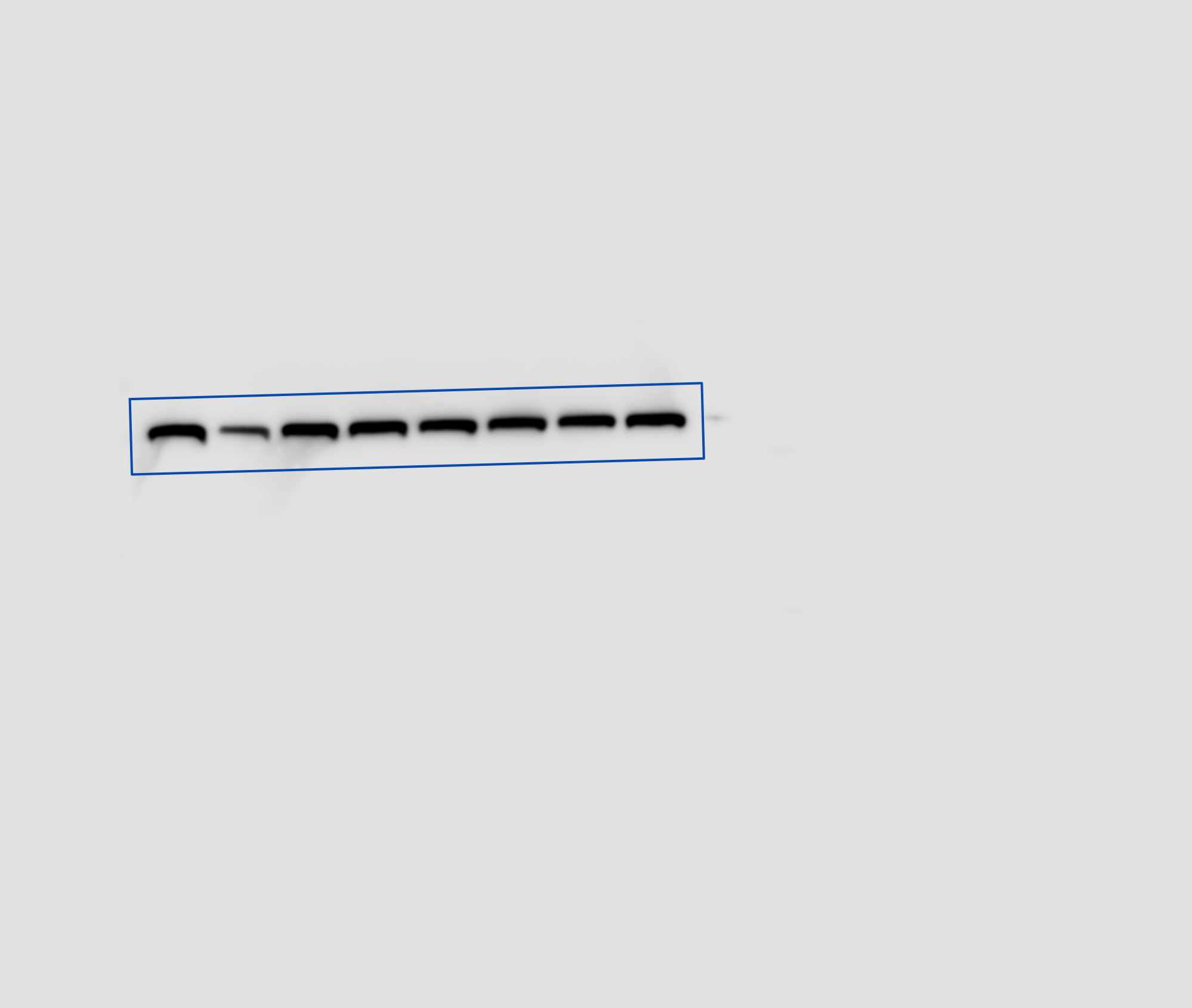

Supplement: Source data 1. [file elife-78609-data1.zip › WB TIFF images - source data - relevant bands/Figure 4A - actin SDS - relevant bands.png]

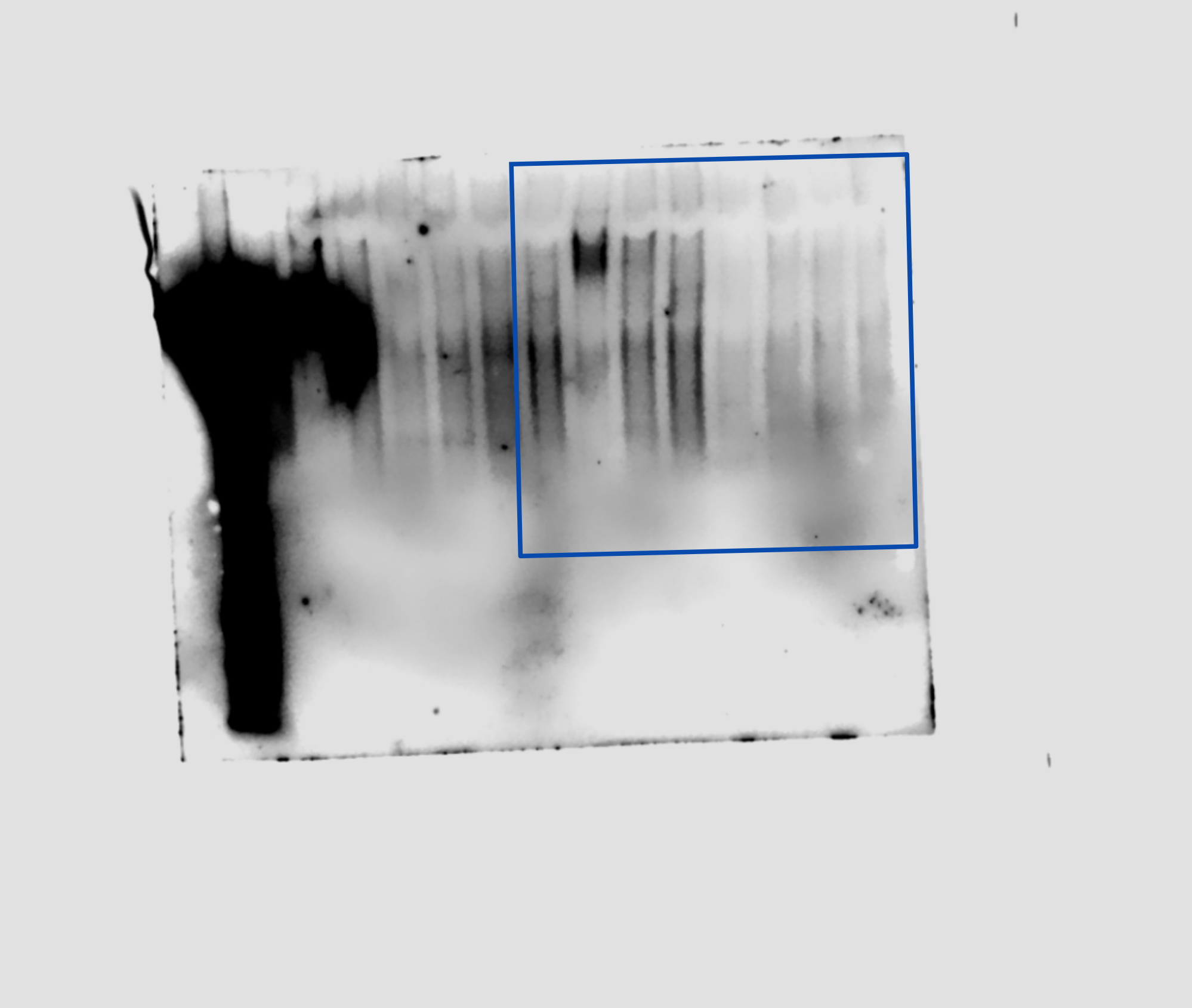

Supplement: Source data 1. [file elife-78609-data1.zip › WB TIFF images - source data - relevant bands/Figure 4A - native NINJ1 - relevant bands.png]

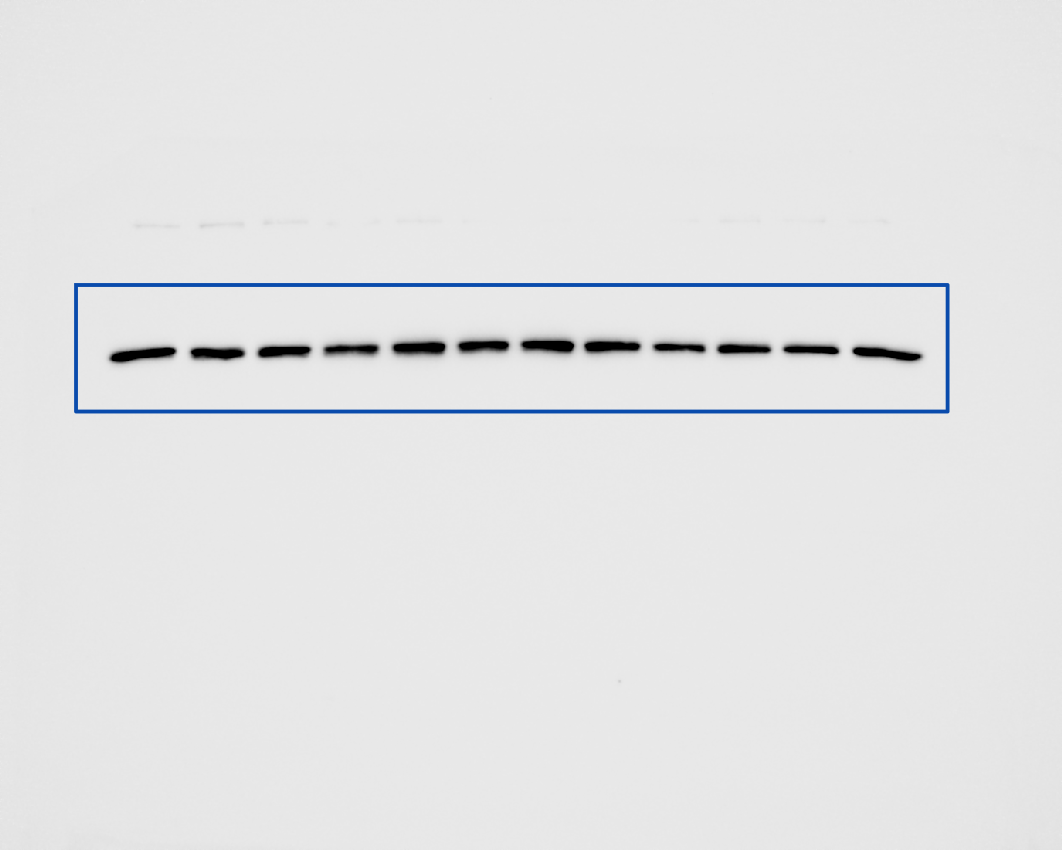

Supplement: Source data 1. [file elife-78609-data1.zip › WB TIFF images - source data - relevant bands/Figure 5 - figure supplement 1A - actin - relevant bands.png]

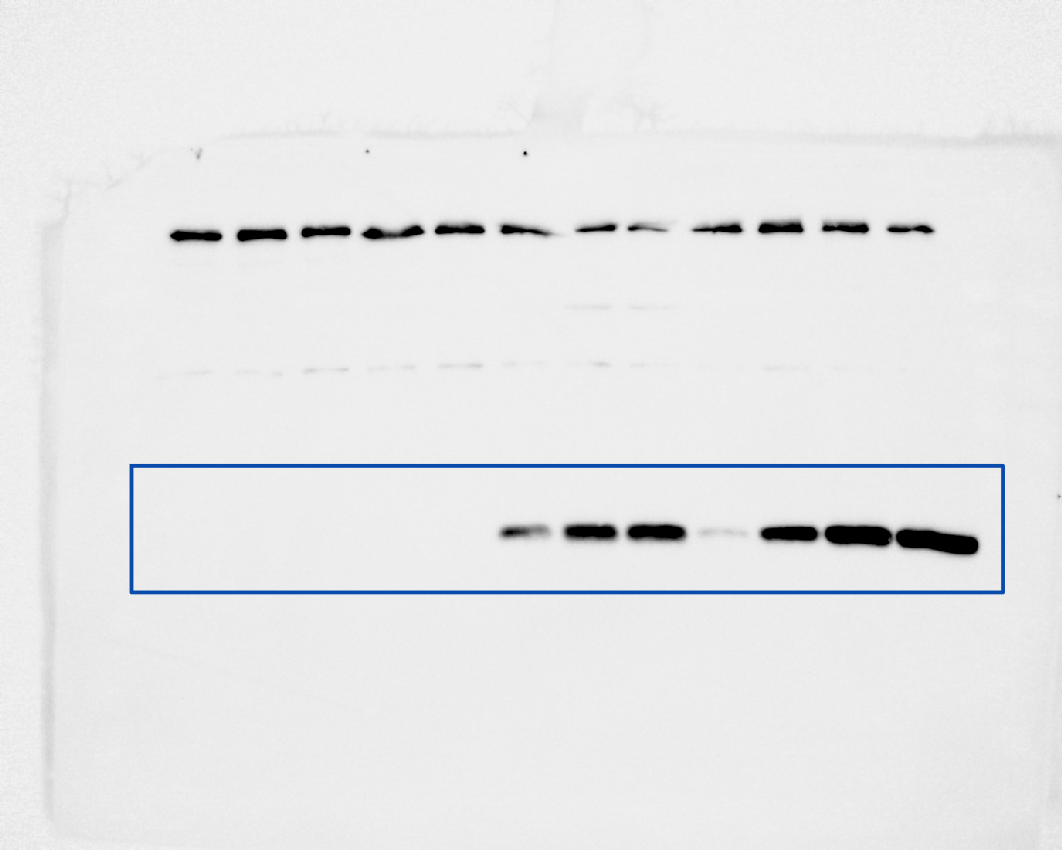

Supplement: Source data 1. [file elife-78609-data1.zip › WB TIFF images - source data - relevant bands/Figure 5 - figure supplement 1A - FLAG - relevant bands.png]

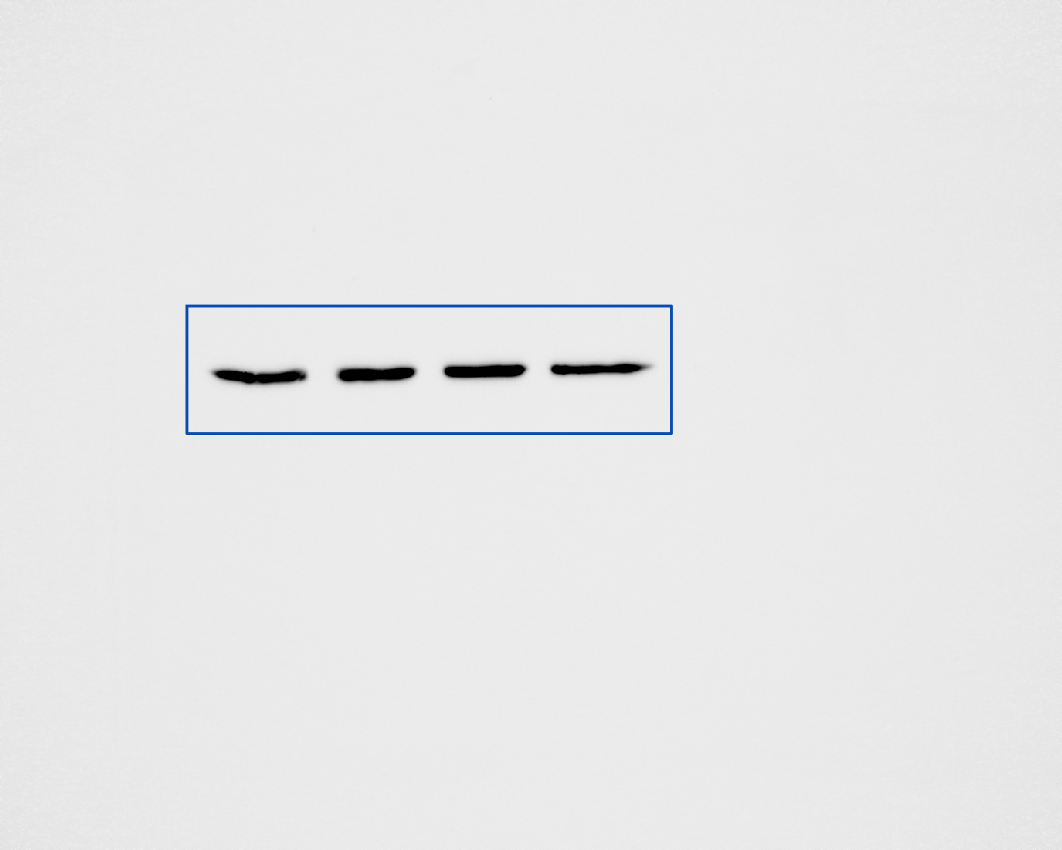

Supplement: Source data 1. [file elife-78609-data1.zip › WB TIFF images - source data - relevant bands/Figure 5A - actin - relevant bands.png]

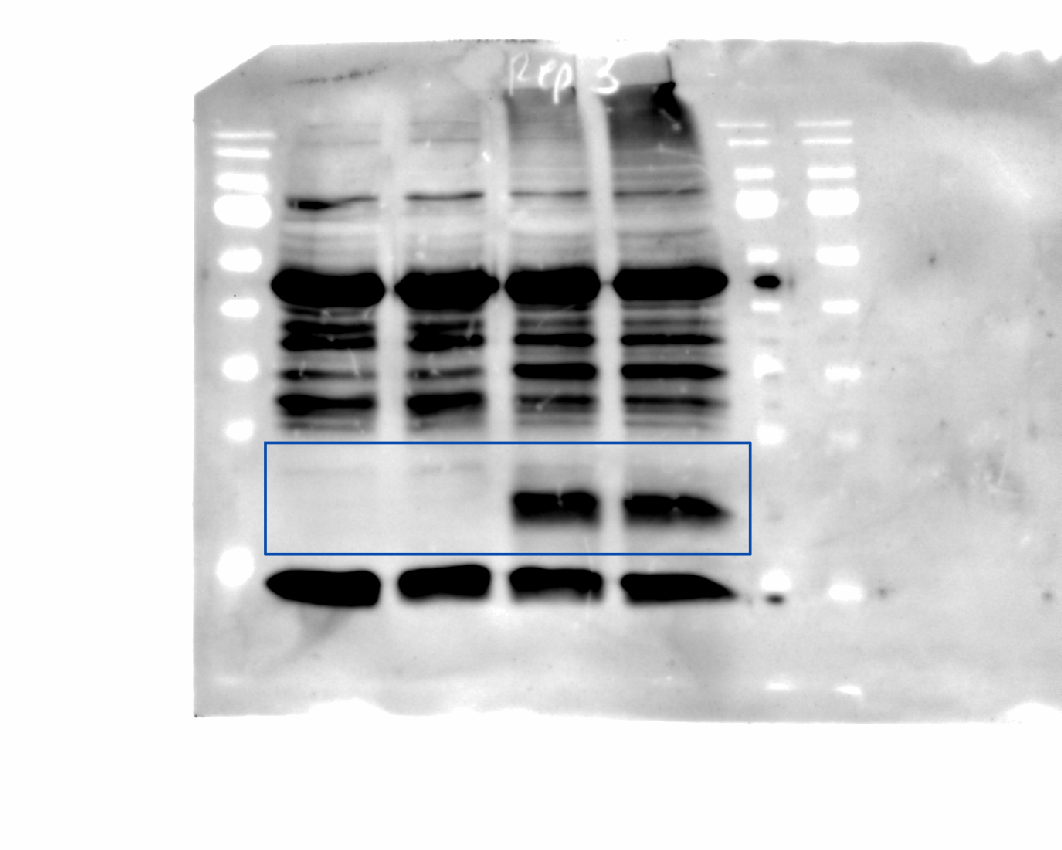

Supplement: Source data 1. [file elife-78609-data1.zip › WB TIFF images - source data - relevant bands/Figure 5A - casp1 p20 - relevant bands.png]

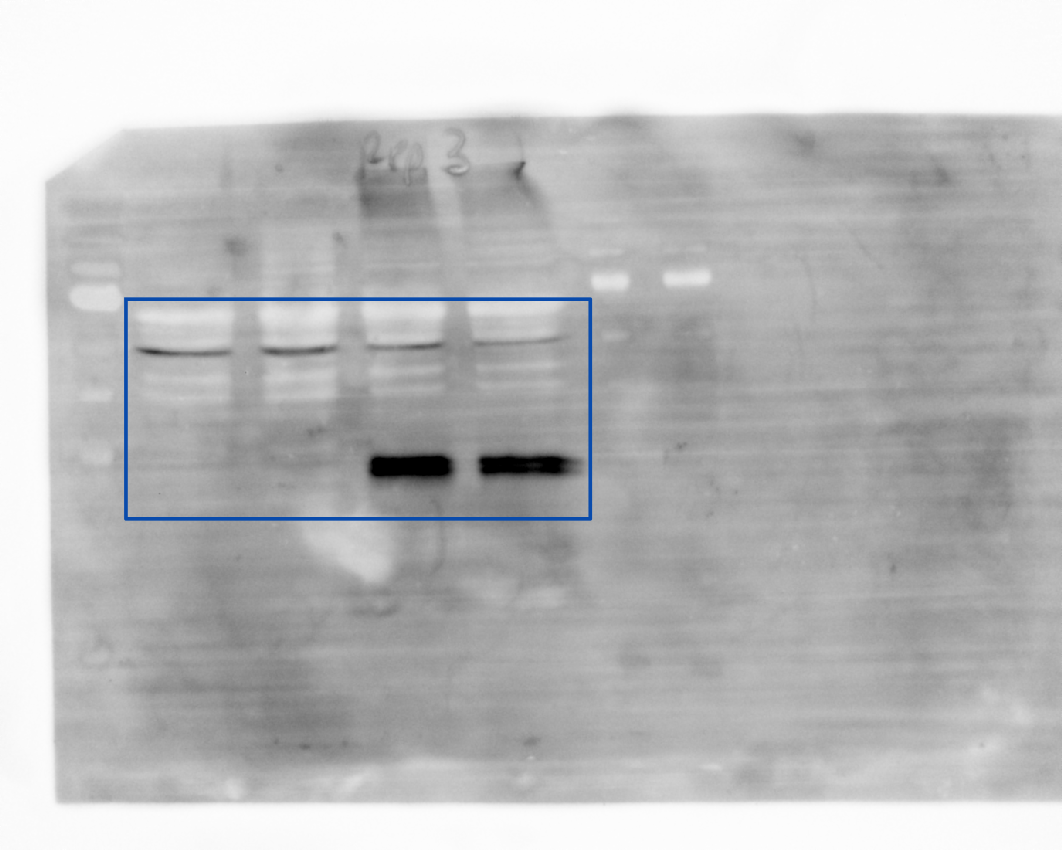

Supplement: Source data 1. [file elife-78609-data1.zip › WB TIFF images - source data - relevant bands/Figure 5A - GSDMD - relevant bands.png]

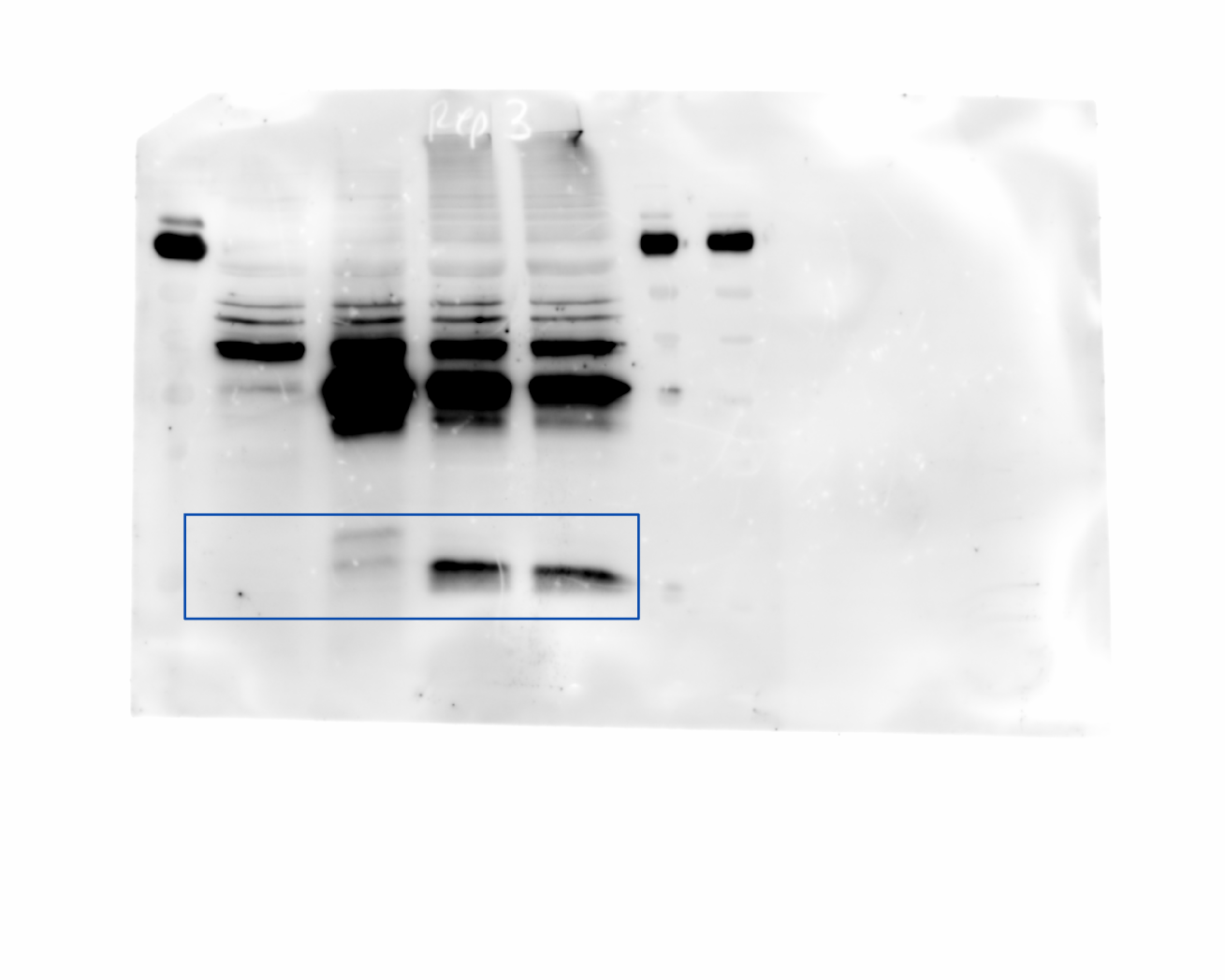

Supplement: Source data 1. [file elife-78609-data1.zip › WB TIFF images - source data - relevant bands/Figure 5A - IL1b - relevant bands.png]

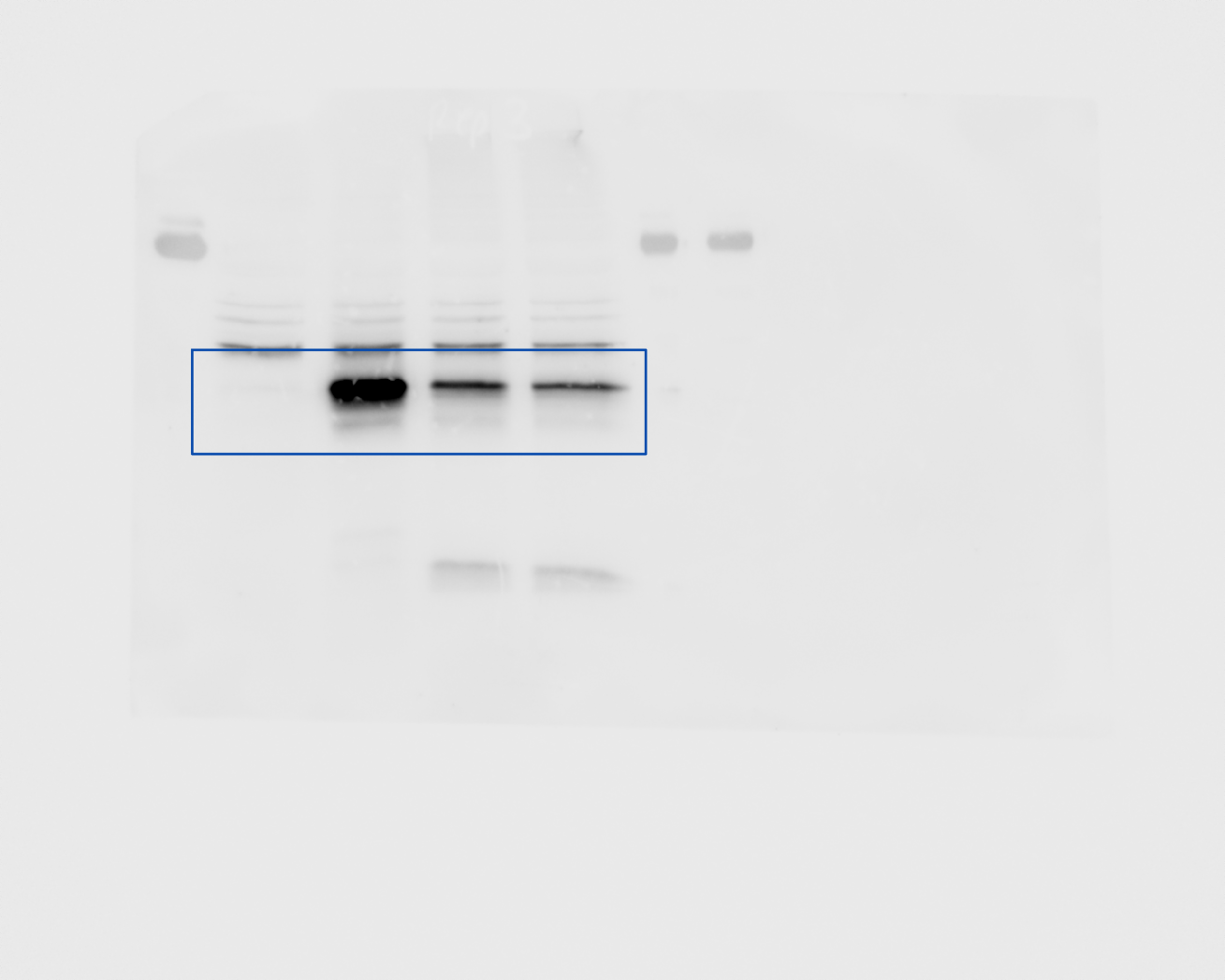

Supplement: Source data 1. [file elife-78609-data1.zip › WB TIFF images - source data - relevant bands/Figure 5A - proIL1b - relevant bands.png]

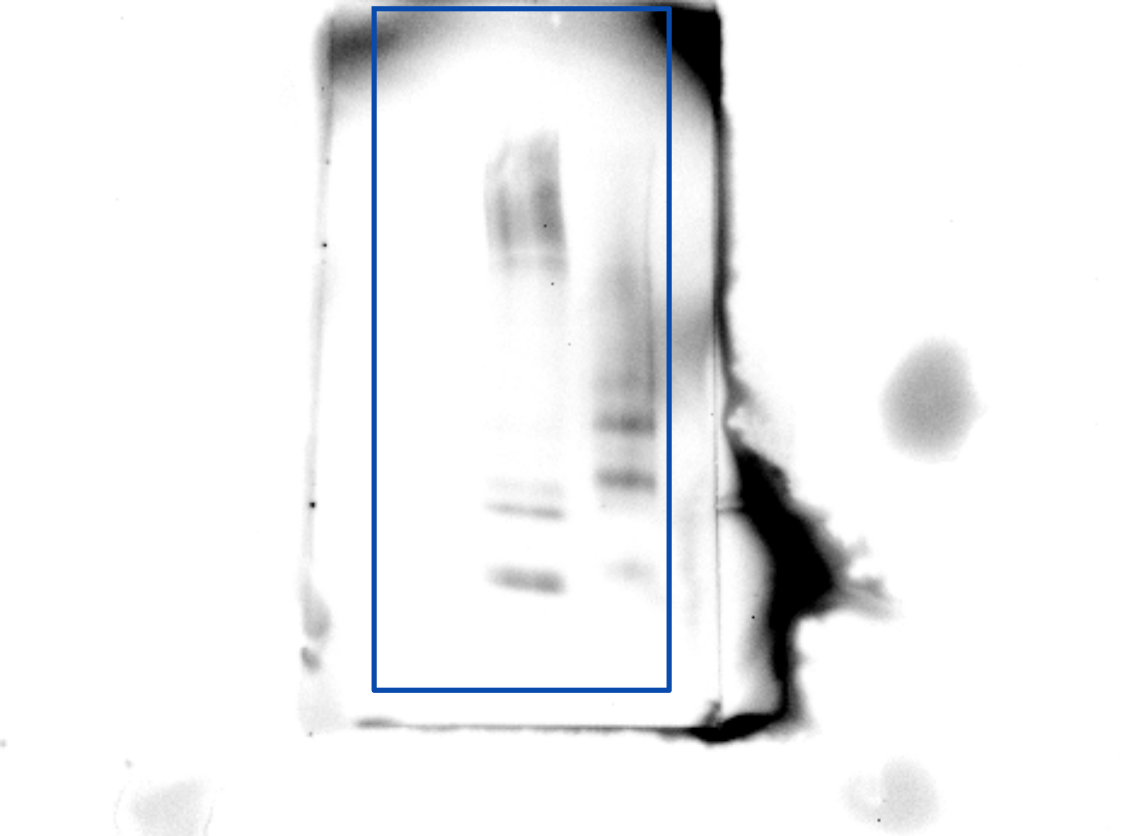

Supplement: Source data 1. [file elife-78609-data1.zip › WB TIFF images - source data - relevant bands/Figure 5E - native HA-NINJ1 - relevant bands.png]

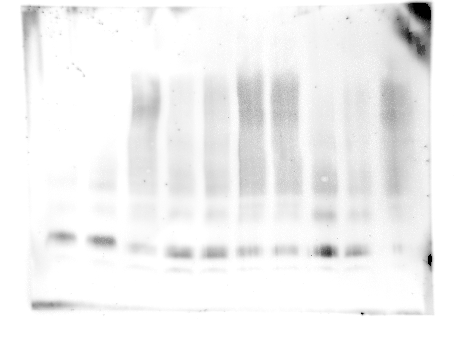

Supplement: Source data 2. [file elife-78609-data2.zip › WB TIFF images - source data/Figure 1 - figure supplement 1C - native NINJ1.tif]

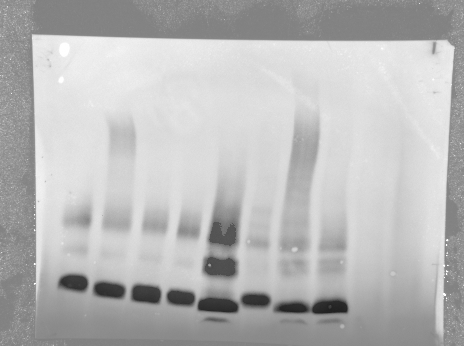

Supplement: Source data 2. [file elife-78609-data2.zip › WB TIFF images - source data/Figure 1 - figure supplement 2D - native NINJ1 col.tif]

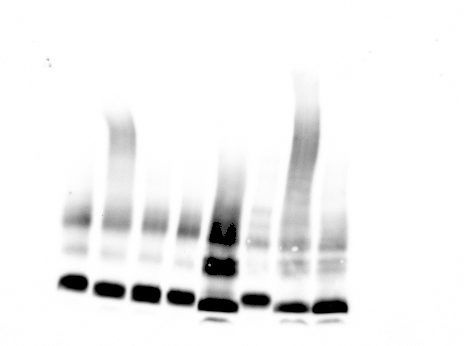

Supplement: Source data 2. [file elife-78609-data2.zip › WB TIFF images - source data/Figure 1 - figure supplement 2D - native NINJ1.tif]

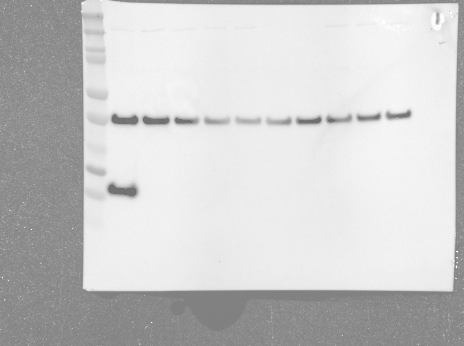

Supplement: Source data 2. [file elife-78609-data2.zip › WB TIFF images - source data/Figure 1 suppl 2A -- GAPDH (1).tif]

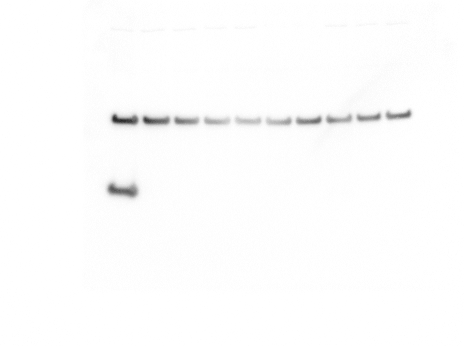

Supplement: Source data 2. [file elife-78609-data2.zip › WB TIFF images - source data/Figure 1 suppl 2A -- GAPDH (2).tif]

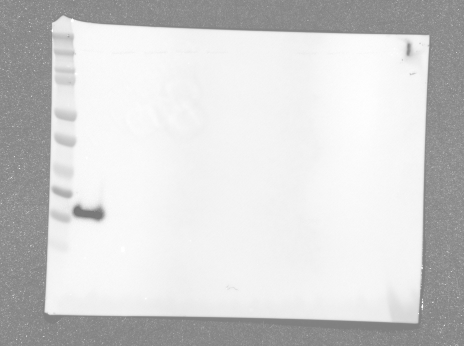

Supplement: Source data 2. [file elife-78609-data2.zip › WB TIFF images - source data/Figure 1 suppl 2A -- NINJ1 (1).tif]

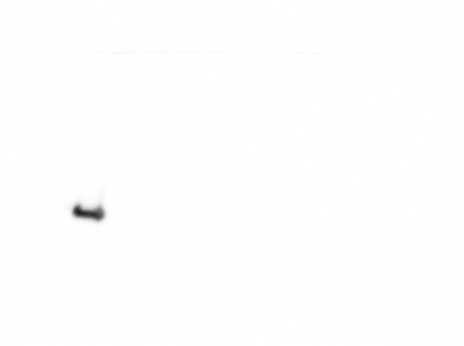

Supplement: Source data 2. [file elife-78609-data2.zip › WB TIFF images - source data/Figure 1 suppl 2A -- NINJ1 (2).tif]

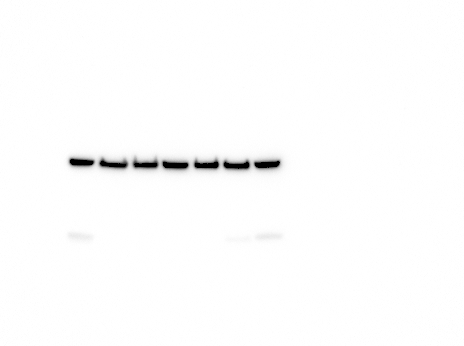

Supplement: Source data 2. [file elife-78609-data2.zip › WB TIFF images - source data/Figure 1A - iBMDM GAPDH.tif]

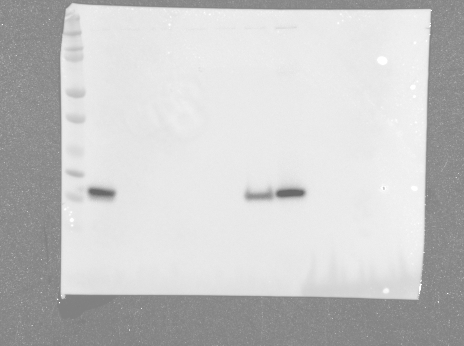

Supplement: Source data 2. [file elife-78609-data2.zip › WB TIFF images - source data/Figure 1A - iBMDM NINJ1 KO clones (1).tif]

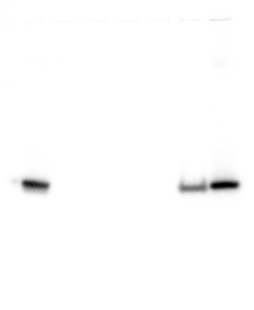

Supplement: Source data 2. [file elife-78609-data2.zip › WB TIFF images - source data/Figure 1A - iBMDM NINJ1 KO clones (2).tif]

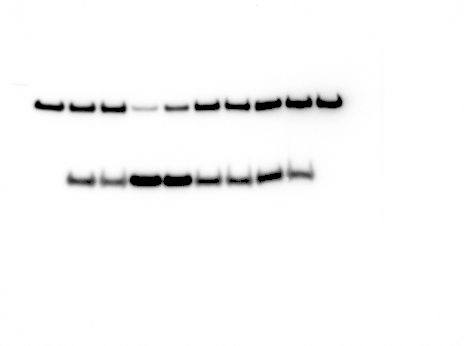

Supplement: Source data 2. [file elife-78609-data2.zip › WB TIFF images - source data/Figure 3A - NINJ1 GAPDH SDS.tif]

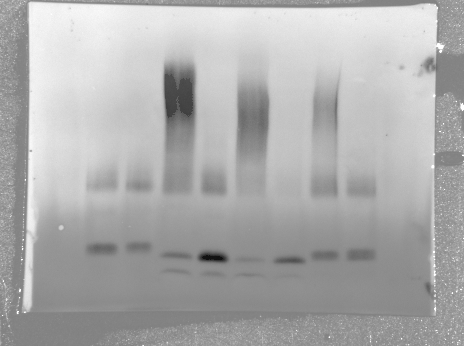

Supplement: Source data 2. [file elife-78609-data2.zip › WB TIFF images - source data/Figure 3A - NINJ1 native page colorimetric.tif]

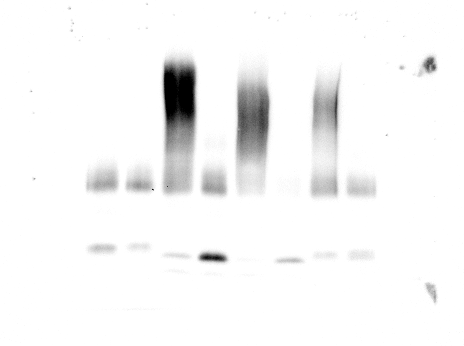

Supplement: Source data 2. [file elife-78609-data2.zip › WB TIFF images - source data/Figure 3A - NINJ1 native.tif]

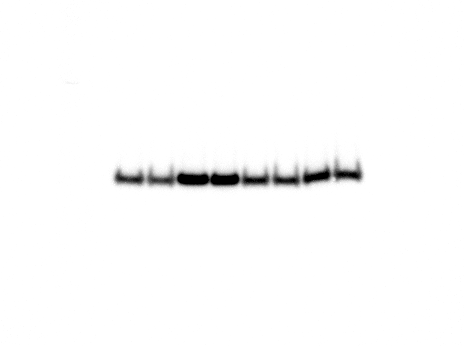

Supplement: Source data 2. [file elife-78609-data2.zip › WB TIFF images - source data/Figure 3A - NINJ1 SDS.tif]

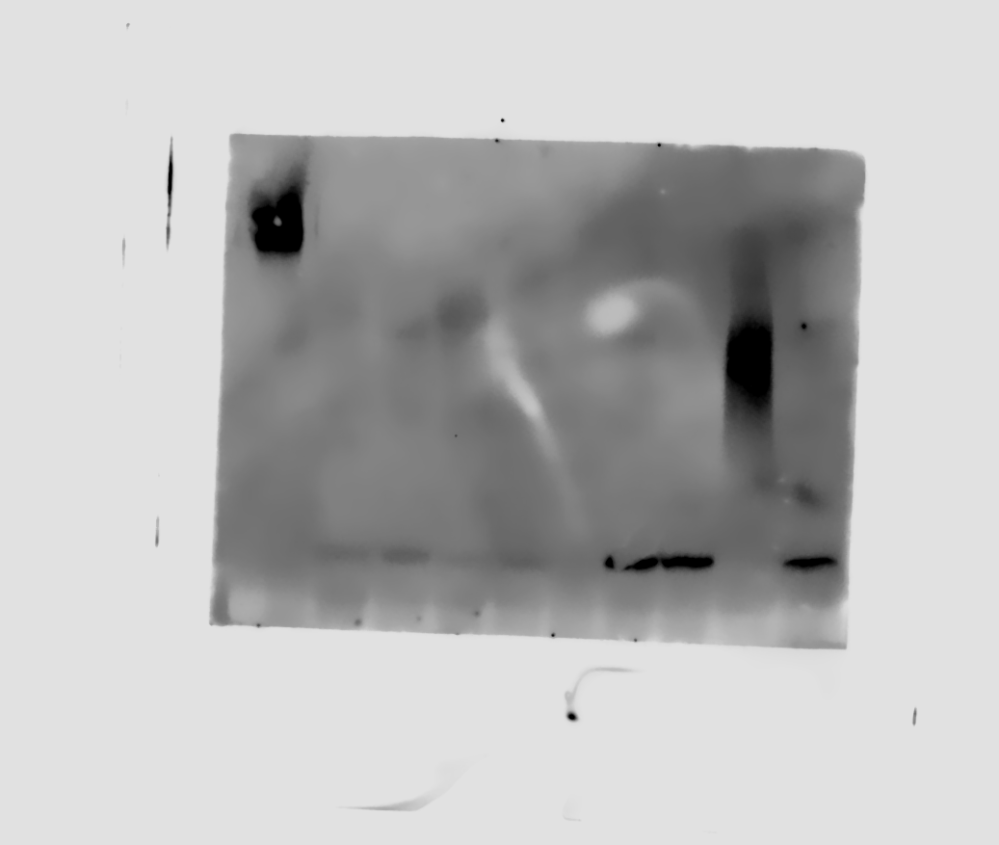

Supplement: Source data 2. [file elife-78609-data2.zip › WB TIFF images - source data/Figure 4 suppl2B Native iPSDM.tif]

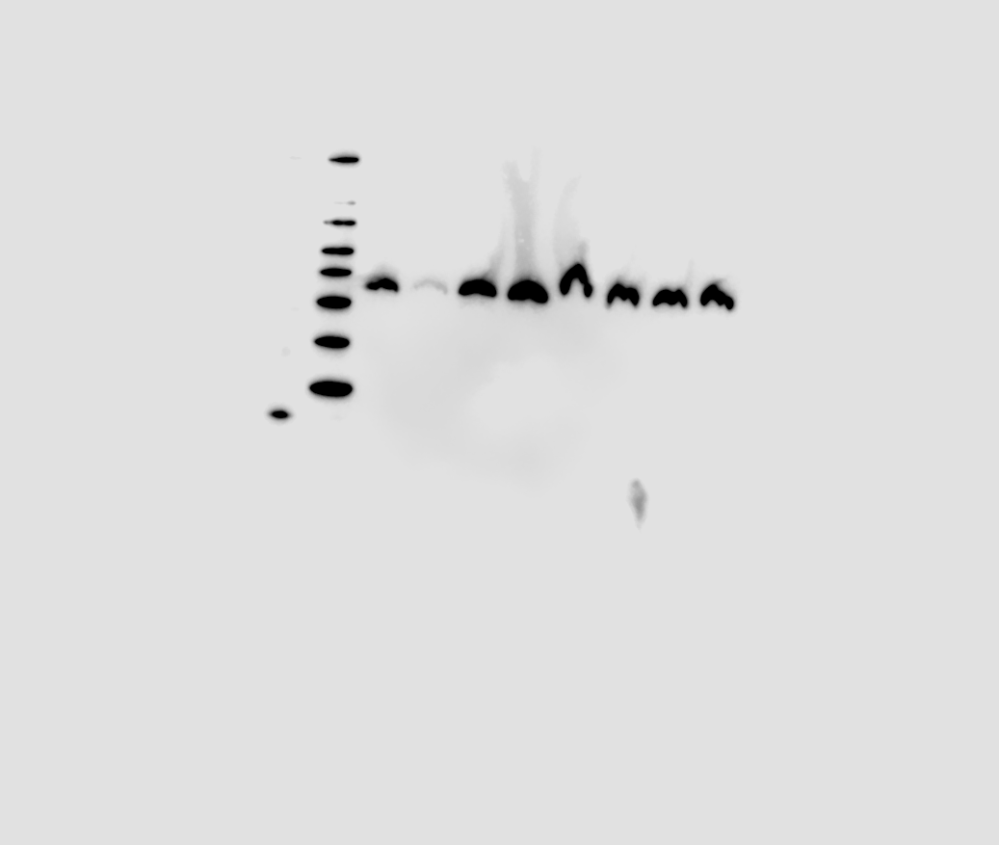

Supplement: Source data 2. [file elife-78609-data2.zip › WB TIFF images - source data/Figure 4 suppl2B WB iPSDM B-actin.tif]

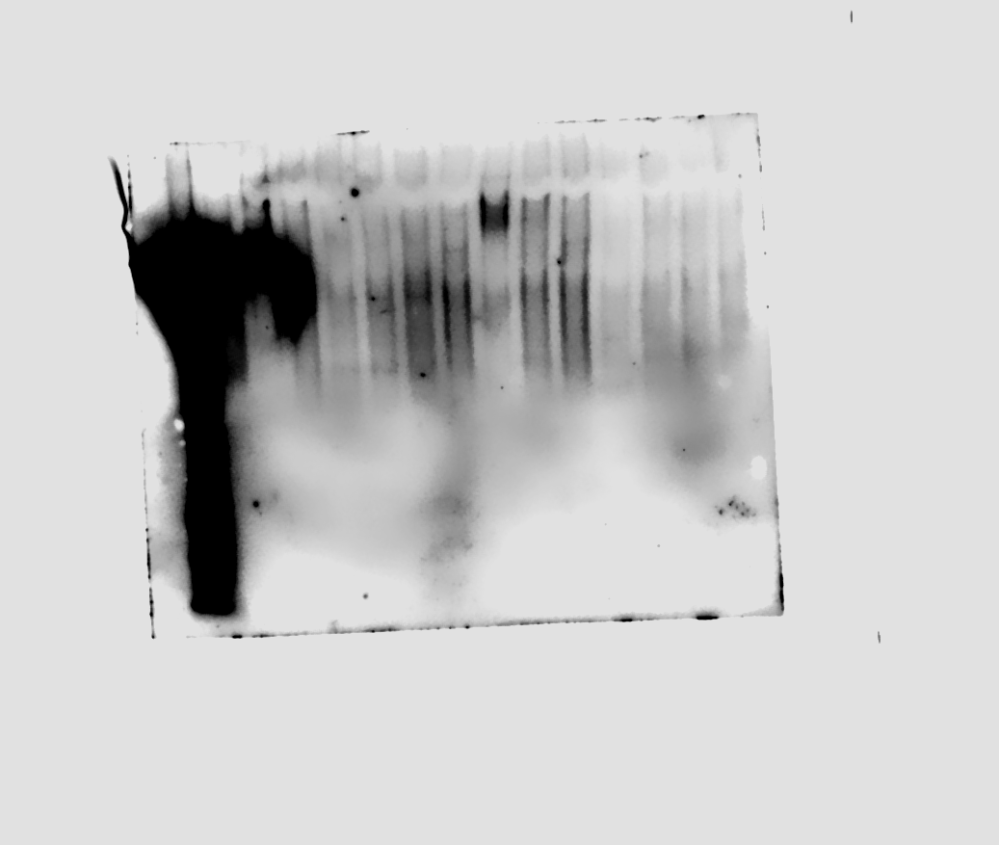

Supplement: Source data 2. [file elife-78609-data2.zip › WB TIFF images - source data/Figure 4A Native MDMs NINJ1.tif]

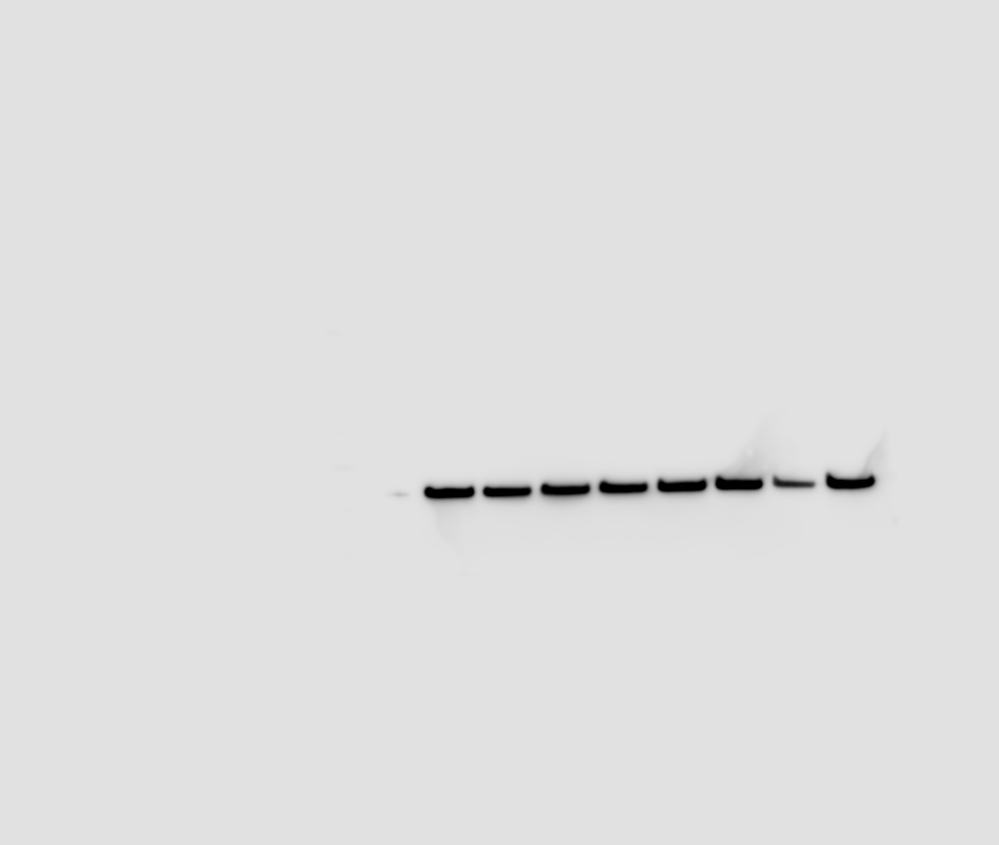

Supplement: Source data 2. [file elife-78609-data2.zip › WB TIFF images - source data/Figure 4A SDS MDM B-actin.tif]

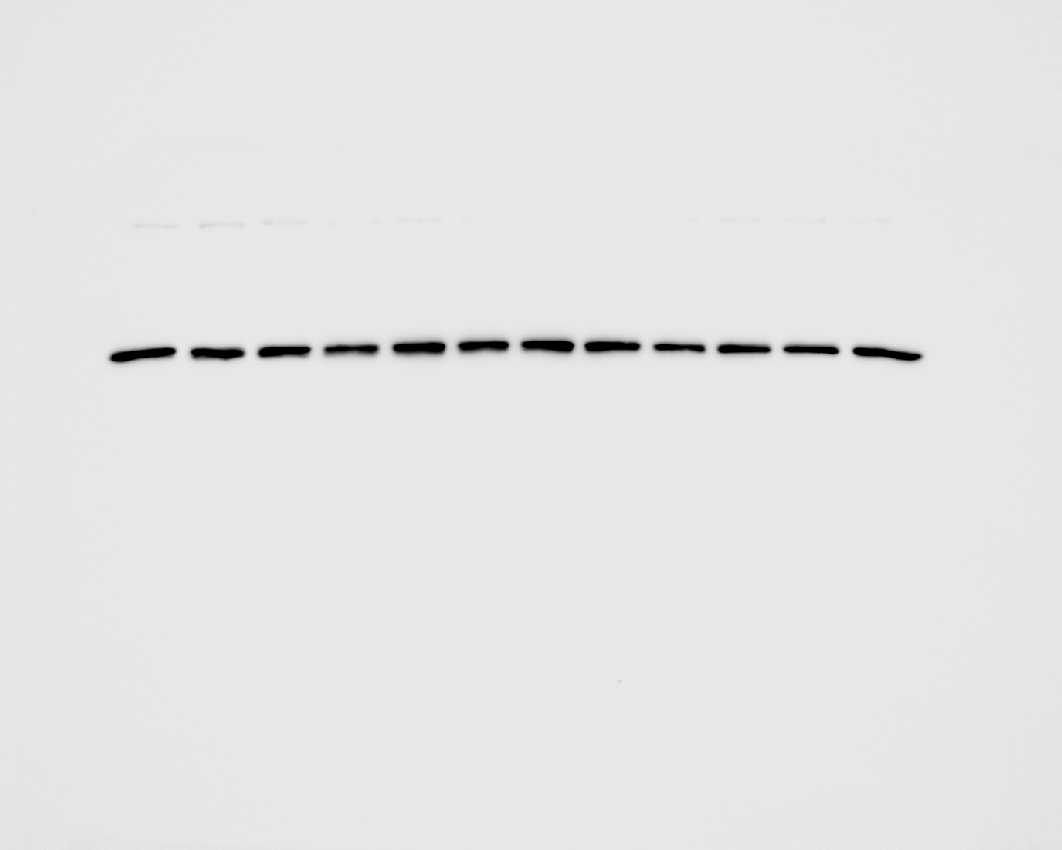

Supplement: Source data 2. [file elife-78609-data2.zip › WB TIFF images - source data/Figure 5 - figure supplement 1A - actin ninj1dox cas9.tif]

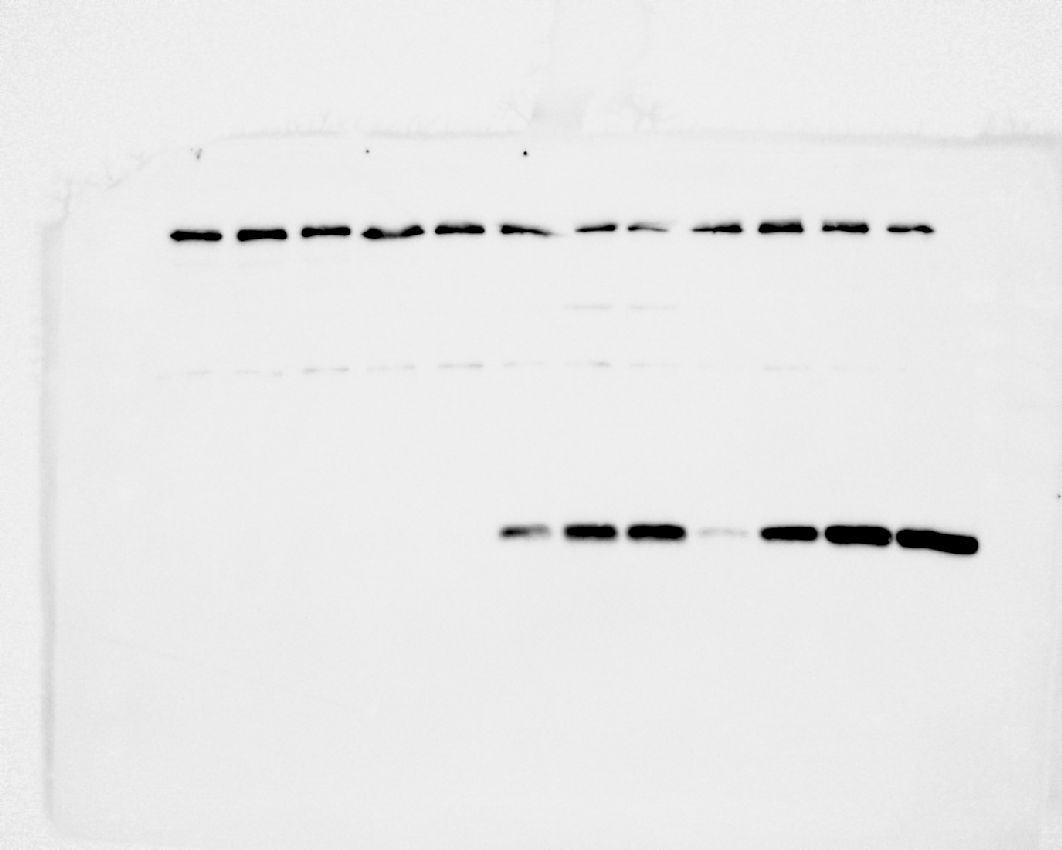

Supplement: Source data 2. [file elife-78609-data2.zip › WB TIFF images - source data/Figure 5 - figure supplement 1A -- flag dox ninj1.tif]

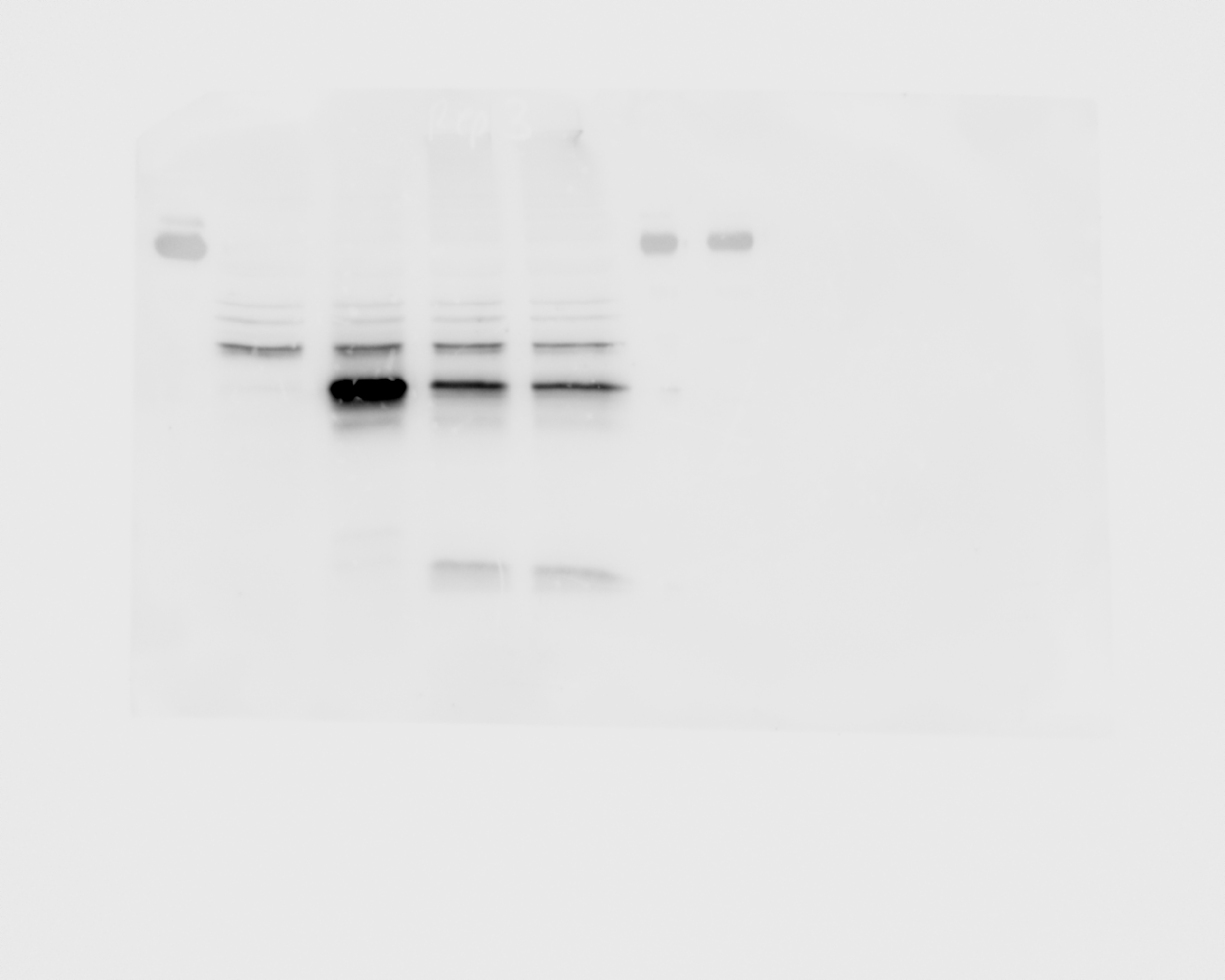

Supplement: Source data 2. [file elife-78609-data2.zip › WB TIFF images - source data/Figure 5A - 2022-07-06 2.tif]

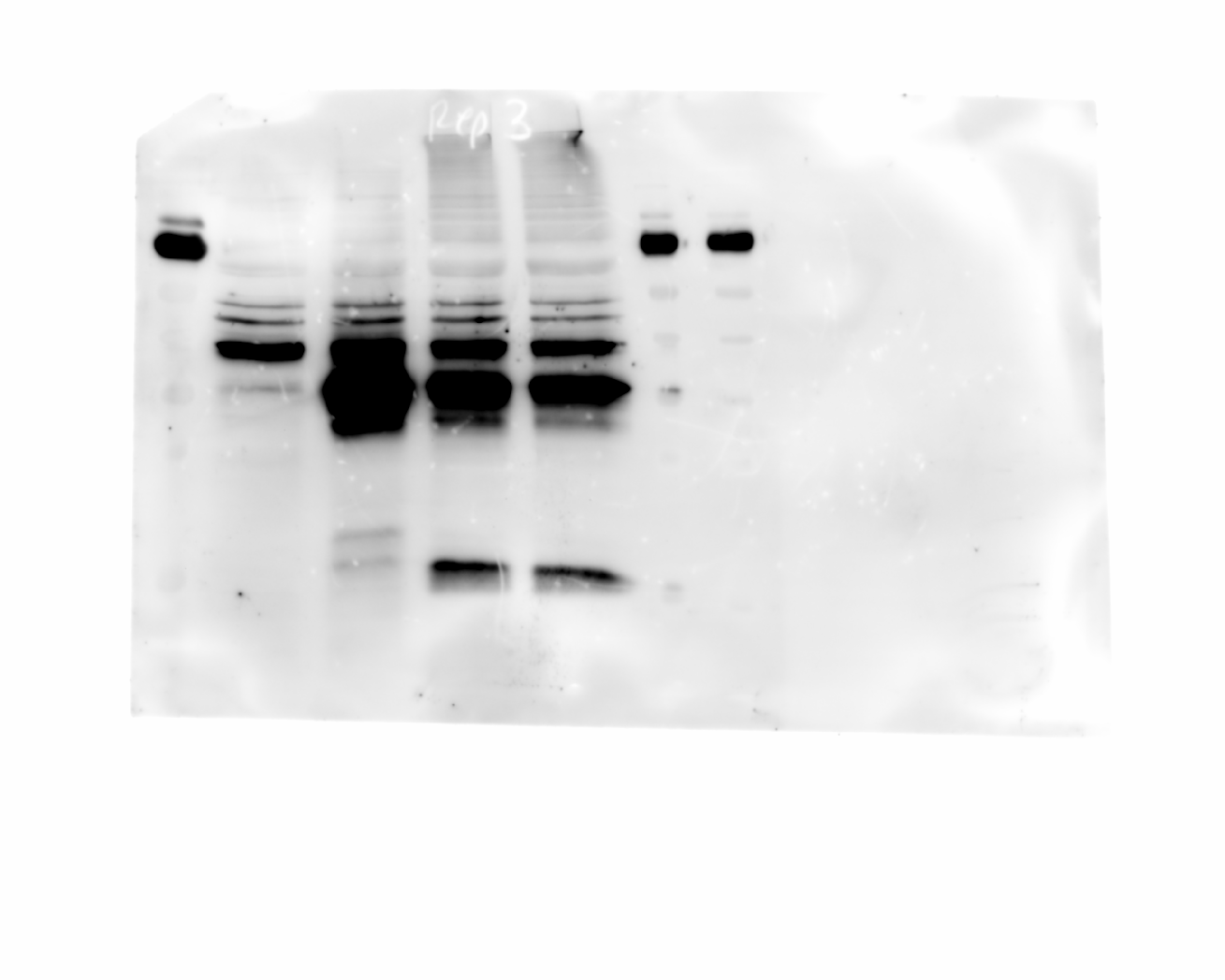

Supplement: Source data 2. [file elife-78609-data2.zip › WB TIFF images - source data/Figure 5A - 2022-07-06.tif]

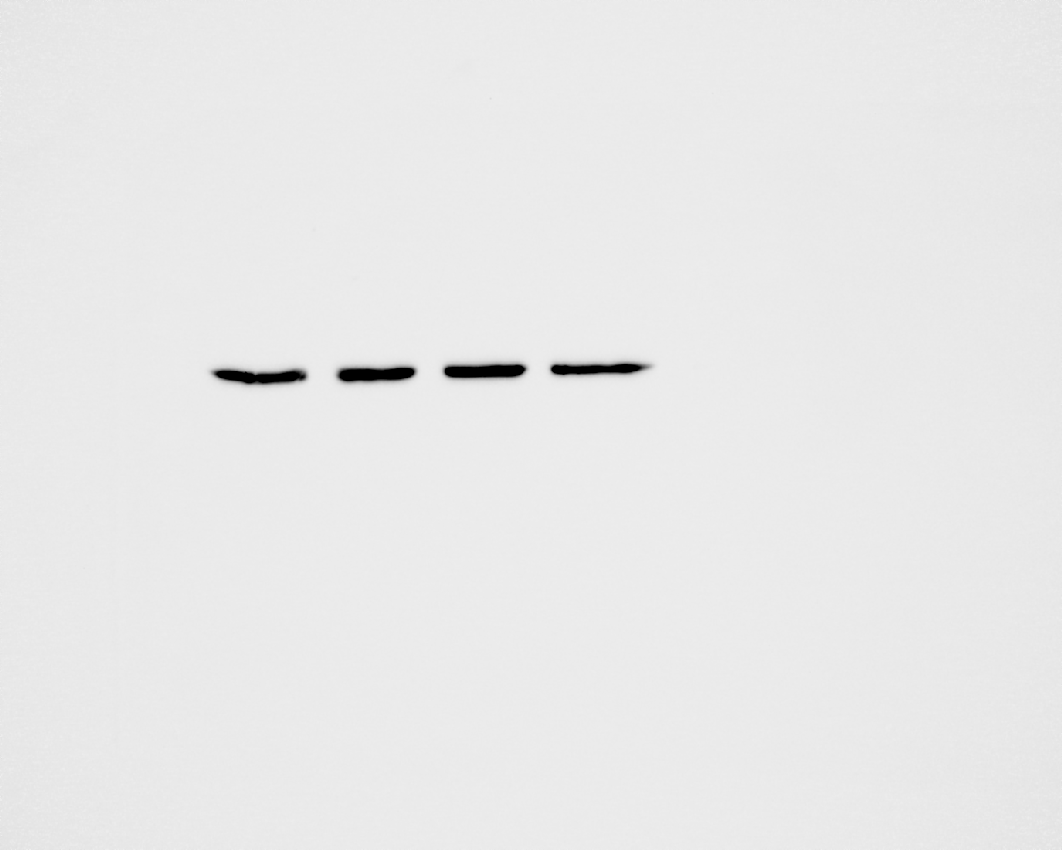

Supplement: Source data 2. [file elife-78609-data2.zip › WB TIFF images - source data/Figure 5A - actin nigericin + gly.tif]

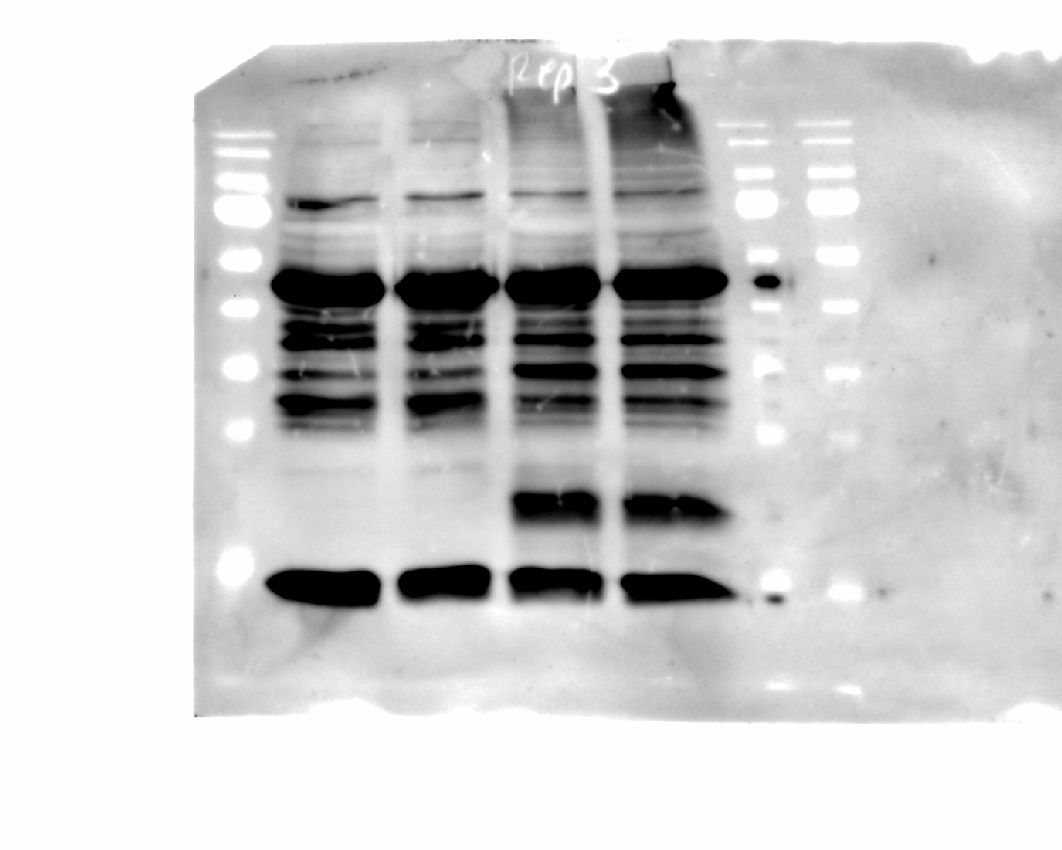

Supplement: Source data 2. [file elife-78609-data2.zip › WB TIFF images - source data/Figure 5A - casp1 cleavage glycine.tif]

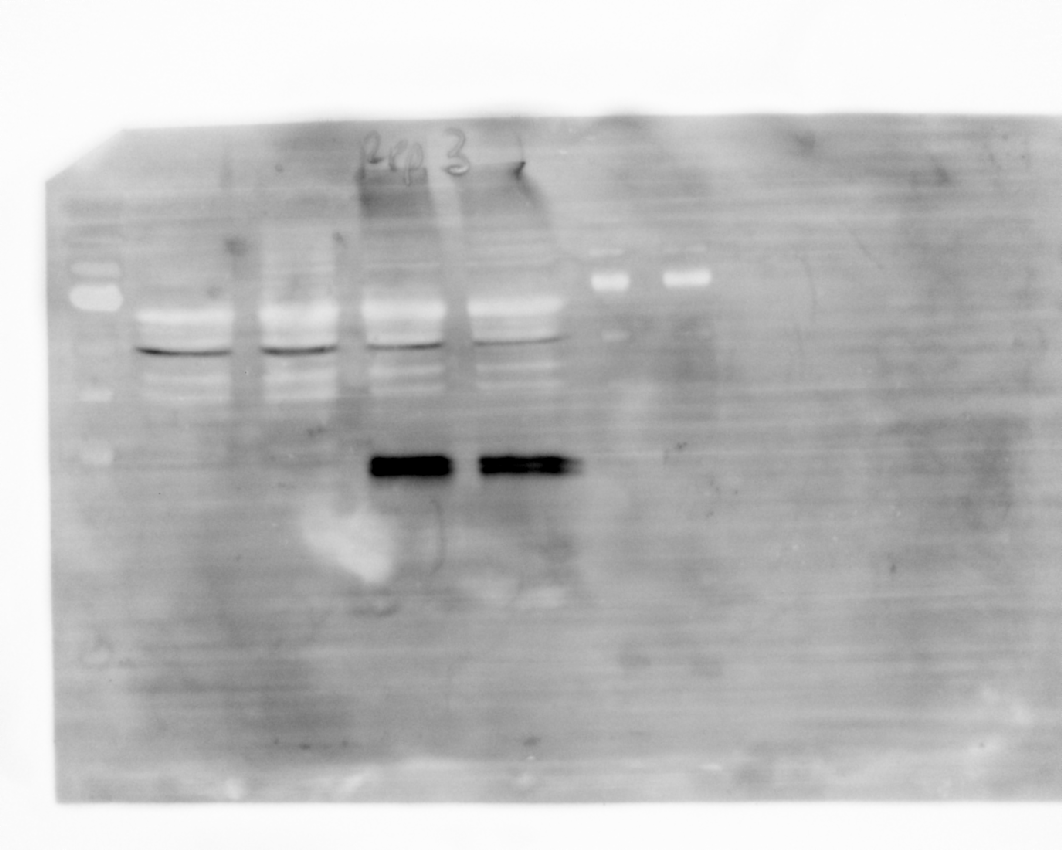

Supplement: Source data 2. [file elife-78609-data2.zip › WB TIFF images - source data/Figure 5A - GSDMD cleavage.tif]

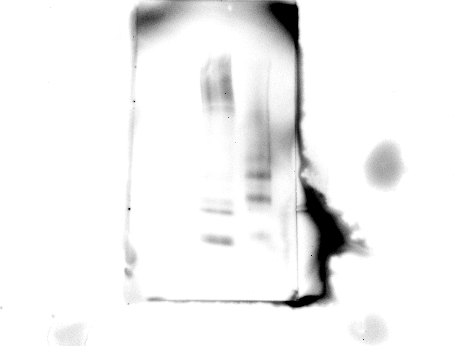

Supplement: Source data 2. [file elife-78609-data2.zip › WB TIFF images - source data/Figure 5F - HA native-PAGE.tif]
